# Dental Data Perform Relatively Poorly in Reconstructing Mammal Phylogenies: Morphological Partitions Evaluated with Molecular Benchmarks

**DOI:** 10.1093/sysbio/syw116

**Published:** 2017-03-14

**Authors:** Robert S. Sansom, Matthew Albion Wills, Tamara Williams

**Affiliations:** 1 *School of Earth and Environmental Sciences, University of Manchester, Oxford Road, Manchester M13 9PT, UK*; 2 *Department of Biology and Biochemistry, University of Bath, Claverton Down, Bath BA2 7AY, UK*

## Abstract

Phylogenetic trees underpin reconstructions of evolutionary history and tests of evolutionary hypotheses. They are inferred from both molecular and morphological data, yet the relative value of morphology has been questioned in this context due to perceived homoplasy, developmental linkage, and nonindependence of characters. Nevertheless, fossil data are limited to incomplete subsets of preserved morphology, and different regions are treated as equivalent. Through meta-analysis of 40 data sets, we show here that the dental and osteological characters of mammals convey significantly different phylogenetic signals, and that osteological characters are significantly more compatible with molecular trees. Furthermore, the application of simplified paleontological filters (retaining only dental data) results in significantly greater loss of phylogenetic signal than random character ablation. Although the mammal fossil record is largely comprised of teeth, dental data alone are generally found to be less reliable for phylogenetic reconstruction given their incongruence with osteological and molecular data. These findings highlight the need for rigorous meta-analyses of distributions of homoplasy in morphological data. These tests, and consequent refinements to phylogenetic analyses that they permit, promise to improve the quality of all macroevolutionary studies that hinge on accurate trees. [Homoplasy; Mammalia; morphology; osteology; phylogeny; teeth

As with all data used to infer phylogeny, morphological characters should be independent, and character states homologous. For molecular sequence data, modeling approaches provide objective tests and qualification of these assumptions. Morphological data, however, are more intractable; anatomical character complexes are subject to comparatively greater levels of developmental and functional linkage, ecological convergence, and subjective interpretation (O’Keefe and Wagner 2001; Scotland 2003; DeGusta 2004; Kangas et al. 2004; Evans et al. 2007; Minelli 2007; Springer et al. 2007; Sadleir and Makovicky 2008; Harjunmaa et al. 2014). Nevertheless, morphology can be hugely informative under many circumstances. Not only can it increase resolution and reveal hidden support (Gatesy et al. 1999; Gatesy and Baker 2005), but because it is usually the only class of data available from fossils, it is the best way to address historic patterns, break up long branches, and calibrate molecular clocks (Wiens 2004; Cobbett et al. 2007; Benton et al. 2005). It is, therefore, necessary to know whether some classes of morphology are more reliable than others. A better understanding of the distribution of homoplasy across different character types would enable more sophisticated treatments of morphological data and would be especially beneficial where the decisiveness of data is poor.

Here we focus on the dental and osteological data of mammals. Dental data have been used alone and alongside other morphological data to reconstruct a wide range of evolutionary transitions from the radiation of mammals (Bi et al. 2014), to the relationships of human ancestors (Strait and Grines 2004). The focus and reliance on dental data are necessitated in large measure by the taphonomic filter particular to the group; the enhanced preservation potential of mammal teeth relative to bones means that many fossil species are known only from dental data. This makes testing for differences between readily fossilizable data (teeth) and less-fossilizable data (osteology) a priority because taphonomic biases can systematically undermine evolutionary inferences (Sansom et al. 2010, 2011, 2013, 2014; Sansom and Wills 2013). In addition to that, particular concerns about character linkage and overall levels of homoplasy in teeth have been raised on several grounds. Studies of tooth development in mice have revealed dramatic and correlated changes in the number and shape of cusps resulting from minor manipulation of developmental pathways (Kangas et al. 2004). This suggests that dental characters are nonindependent and this is supported by subsequent developmental studies focusing on phenotypes and traits (Harjunmaa et al. 2014). Furthermore, measures of phenotypic complexity indicate that the morphology of mammal teeth is strongly tied to function such that form is highly homoplastic and contingent on diet (Evans et al. 2007). Aside from developmental and functional linkage, comparison of levels of dissimilarity in molecular and dental characters of phyllostomid bats found the latter to be oversaturated and potentially nonindependent (Dávalos et al. 2014), with the inference that this problem could be more widespread. Nondental anatomy is also expected to exhibit some degree of nonindependence and linkage; for example, serial homology is observed in tetrapod limbs (e.g., Ruvinksy and Gibson-Brown 2000; Young and Hallgrímsson 2005). Nevertheless, it is mammal teeth for which developmental linkage of phylogenetic characters has been empirically demonstrated (Harjunmaa et al. 2014).

Investigations of morphological partitions of empirical data (dental, cranial, and postcranial) have found similar levels of homoplasy in some cases (e.g., Sánchez-Villagra andWilliams 1998; Mounce et al. 2016). As such, the characters partitions have been interpreted as equally informative. The same is true for isolated studies of primates (Williams 2007) and Cetartiodactylia (O’Leary et al. 2003), although an earlier analysis of the latter’s data found teeth to be “markedly different from the rest of the morphological data” (Naylor and Adams 2001, p. 451) [see O’Leary et al. (2003) and Naylor and Adams (2001) for further discussion]. This raises a number of questions: Why is there an apparent discordance between developmental studies and empirical morphological data regarding the relative phylogenetic informativeness of tooth morphology? Are there differences between the phylogenetic signals contained within dental and osteological morphology? Can dental data alone be used reliably to reconstruct the evolutionary history of mammals? To answer these questions we compiled morphological data sets comprising dental and osteological characters for a broad range of mammal clades, both extant and extinct. Rather than use a single data set as a case study, we take a meta-analytical approach to maximize statistical power, maximize taxonomic coverage, and identify broad-scale patterns. While there are alternative partitions that might be applied to partitions of morphological data [e.g., cranial vs. postcranial (Mounce et al. 2016), axial vs. appendicular], our focus on dental versus osteological characters allows us to address specific concerns raised regarding the developmental and functional linkage and oversaturation of dental characters, and the taphonomic biases particular to mammals (i.e., the enhanced preservation potential of enamel over bone).

It is impossible to know the evolutionary history of empirical taxa with certainty, but congruence between trees inferred from different sources of data offers a means for cross-validation. In this context, we used trees derived from molecular data to assess the congruence of different classes of morphological data. Sequence data provide a qualitatively different and vastly larger data source that serves as a suitable and well-validated benchmark. No single case study is compelling, but meta-analyses of combined data from different sources, clades, and authors seek broad-scale patterns and generalizations from a statistically meaningful sample of independent data (e.g., Pisani et al. 2007). We, therefore, use a meta-analytical approach to address the following three questions:

(1) Do morphological partitions of dental and osteological characters convey a homogenous phylogenetic signal? More specifically, we test the null hypothesis that the partitions do not exhibit significant partition heterogeneity according to the incongruence length difference test (ILD: Farris et al. 1995a, 1995b) and incongruence relationships difference test (IRD: Mounce et al. 2016).
(2) Are dental and osteological partitions of morphological data sets equally consistent with trees derived from independent molecular sequence data? The specific null hypothesis is that dental and osteological partitions do not retain different levels of relative homoplasy as assessed by the ensemble retention index (RI: Farris 1989).
(3) Do the generalized taphonomic filters that occur during fossilization (i.e., loss of osteological data, retention of dental data) degrade the phylogentic signal any more or less than equivalent random filters? Specifically, are a similar number of nodes recovered by matrices missing osteological characters compared with matrices missing identical amounts of random characters (as assessed by the node recovery test Sansom and Wills 2013).

## MATERIALS AND METHODS

Morphological data matrices of mammals were compiled from published sources (*Google* and *Google Scholar* searches for “*clade* phylogeny $+$/$-$ morphology” and references and citations therein, and *MorphoBank* (O’Leary and Kaufman 2011) from August 2013 to April 2015. Characters were categorized as either osteological or dental on the basis of tissue type (i.e., characters relating directly and explicitly to teeth vs. osteological tissues such as the mandible). Soft tissue characters were excluded. To ensure a balance in the distribution of missing data, taxa with greater than 50% missing entries in either partition were removed, and data sets with a difference of greater than 10% missing entries between partitions were edited further by removing characters with high proportions of missing data (after Sansom and Wills 2013). All uninformative characters were removed. Thresholds were set for the minimum data set dimensions (10 taxa, 30 characters) and minimum percentage of characters in the smallest partition (20%; Sansom and Wills 2013). To ensure independence of data, data sets with appreciable taxonomic overlap were eliminated by prioritizing the more recent source. Rejected data sets, and the reasons for their rejection, are listed in the Supplementary Materials available on Dryad at http://dx.doi.org/10.5061/dryad.k23mq.

**Figure 1.**
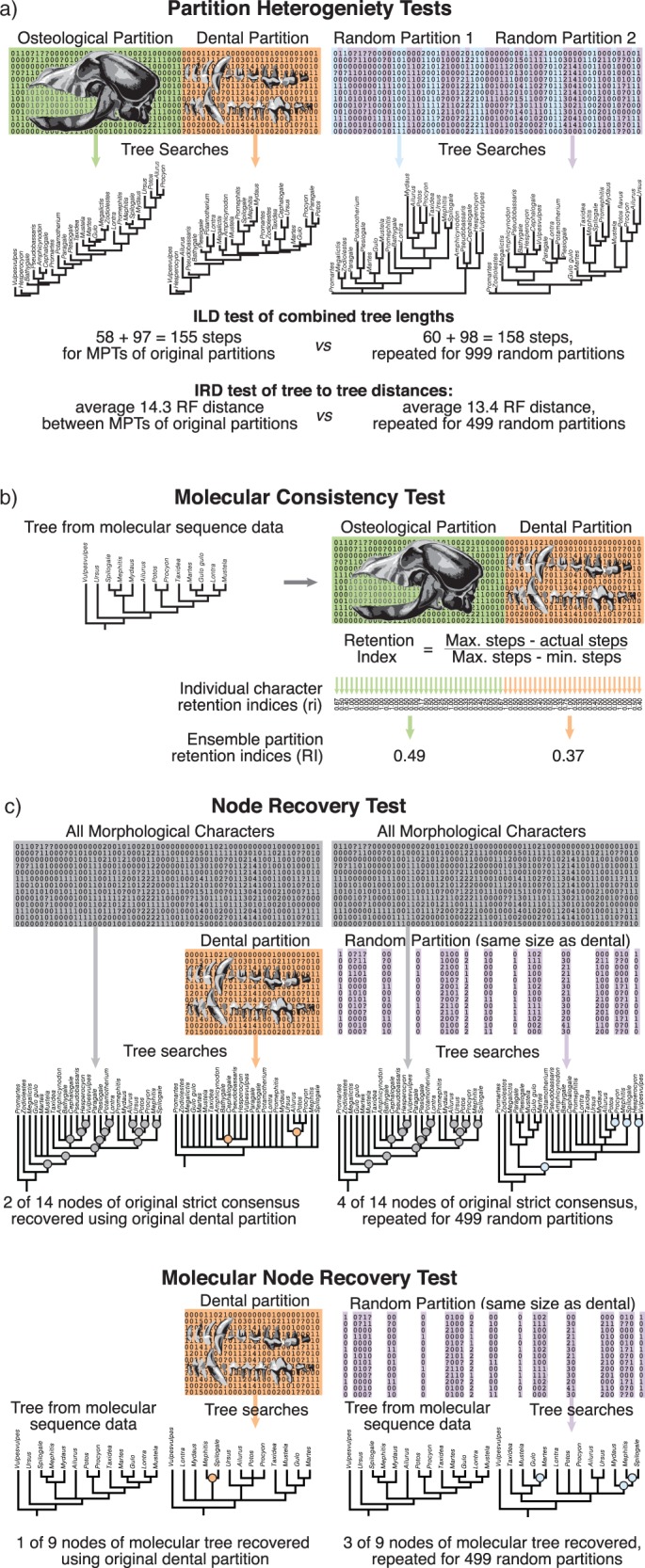
Analyses applied to morphological data. a) Partition heterogeneity tested by comparing combined tree length (ILD) or nearest neighbor tree-to-tree distances of trees (IRD) resulting from searches of each morphological character partition (osteological and dental) and random character partitions of the same size (Farris et al. 1995a, 1995b; Mounce et al. 2016). b) Molecular consistency tested by applying morphological data to a molecular tree and calculating retention indices of characters and partitions. c) Node recovery tested by comparing strict consensus trees resulting from searches using only dental characters and strict consensus trees resulting from searches using random subsets of characters in the same proportion (i.e., loss of signal with either systematic or random character removal). Signal recovery is assessed in terms of nodes shared with either the strict consensus tree using all characters or the molecular tree. Morphological data set example from Finarelli (2008) with Flynn et al. (2005) molecular tree.

We applied the ILD test (Farris et al. 1995a, 1995b; Barker and Lutzoni 2002) to our matrix partitions to test for heterogeneity of signal. The ILD test has the null of partition homogeneity and assesses this by comparing the combined length of shortest trees inferred from each partition to combined lengths of shortest trees inferred from randomly allocated partitions of the same size as the original partitions (Fig. 1a). We used scripts in TNT (Tree analysis with New Technology Goloboff et al. 2008), implementing 999 random replicates. The suitability of the ILD test has been questioned, particularly on the grounds of high type I error rate (false positives) (Dolphin et al. 2000; Hipp et al. 2004; Planet and Sarkarm 2005; Planet 2006; Ramirez 2006; Mounce et al. 2016), but it remains widely used nonetheless. In addition to the ILD test, we implemented the IRD test of Mounce et al. (2016). This is also a randomization-based test, but rather than using differences in inferred tree length, partitions are compared via the distances between the optimal trees that result from them. There are many tree-to-tree distance metrics, but here we used the symmetric difference distance (RF; Robinson and Foulds 1981; Pattengale et al. 2007) for reasons of familiarity and ease of computation. Sets of multiple optimal trees were compared by calculating the mean RF distance between each tree in one set and its nearest neighbor (the nearest neighbor distance) in the other set (Cobbett et al. 2007). As with the ILD test, $P$ values were approximated from 999 random partitions of the data to assess whether the value from the original partitions (either combined lengths of most parsimonious trees or distance between most parsimonious trees) falls significantly outside the range of values observed in random partitions of the same data.

To assess the role of taphonomic biases, we used the node recovery test (Sansom and Wills 2013), also implemented in TNT. The strict consensus trees resulting from maximum parsimony searches using dental characters alone were compared with the strict consensus trees resulting from searches using the entire data set in terms of number shared nodes (i.e., percentage of original signal recovered). This percentage of nodes was then compared with the distribution of percentage of nodes recovered from similar analyses of 500 equivalent matrices, each comprising the same number of characters (i.e., the number of dental characters), but drawn randomly from the entire data set (Fig. 1c).

Molecular trees were sourced in the same way as morphological data. In selecting molecular trees for each morphological data set, priority was given to the degree of taxonomic overlap, and subsequently, the underlying amount of sequence data, rather than the date of publication. Prioritizing taxonomic overlap maximized the data available for analyses because taxa not included in the molecular trees (principally extinct taxa) were removed, and consequently uninformative characters were deleted from all calculations. Both maximum parsimony and likelihood trees were used where available. In the event that multiple trees derived from the same method were presented in a single molecular study, priority was given to the tree derived from the greater amount of sequence data, and subsequently to that which showed greatest resolution. Details of all the molecular trees used are given in the Supplementary Material available on Dryad. Mesquite (Maddison and Maddison 2015) was used to construct trees from published figures and to derive retention indices of morphological characters and partitions. Retention indices are influenced by the data set dimensions and, therefore, cannot be directly compared across matrices. Differences between the partitions were, therefore, assessed using $t$-tests of the ensemble retention index (RI i.e., for the partition as a whole) and mean character retention index (ri i.e., averaged for all characters in a partition), with values from partitions within the same data set paired.

Molecular data were also used for a modified node recovery test (Fig. 1c). Instead of using the strict consensus tree derived from all morphological characters as the baseline for node recovery, a molecular tree was used. This enabled an assessment of whether trees derived using only dental data recovered significantly fewer molecularly compatible nodes than trees derived from a random subset of morphological characters of the same size drawn from across partitions. For this test, taxa for which molecular data were not available were removed, as were the characters subsequently rendered uninformative following the exclusion of those taxa.

## RESULTS

Forty edited data sets (Supplementary Material available on Dryad) were compiled, constituting a combined total of 1234 taxa and 7403 characters and a near comprehensive sampling of the available morphological data for mammals (Table 1).

**Table 1.** Data sets analyzed and partition heterogeneity test values

		Edited matrix		Test $P$ values	
Data source	Ingroup	Taxa	Characters	ILD	IRD
Ahrens (2012)	‡Procyonidae	12	66	0.271	0.467
Asher et al. (2010)	$^{\ast}$ Chrysochloridae	30	115	**0.004**	**0.004**
Asher et al. (2005)	$^{\ast}$ Glires	57	221	**0.004**	0.062
Bi et al. (2014)	$^{\ast}$ Mammalia	37	402	**0.001**	0.114
Billet (2011)	†Notoungulata	41	119	0.072	0.152
Boessenecker and Churchill (2013)	†Odobenidae	17	85	**0.001**	0.090
Boisserie (2005)	†Hippopotamidae	14	36	0.104	0.246
Bryant et al. (1993)	$^{\ast}$ Mustelidae	23	36	**0.024**	**0.042**
Carleton (1980)	$^{\ast}$ Muroidea	71	42	0.443	—
Carstens et al. (2002)	$^{\ast}$ Phyllostomidae	42	62	0.519	**0.019**
Cerdeño (1995)	†Rhinocerotidae	41	71	**0.001**	—
Churchill et al. (2014)	‡Otariidae	24	82	0.064	0.433
Domning (1994)	†Sirenia	31	59	0.073	0.559
Finarelli (2008)	$^{\ast}$ Arctoidea	25	75	**0.001**	**0.006**
Fracasso et al. (2011)	$^{\ast}$ Chiroptera	23	130	0.114	**0.012**
Froehlich (1999)	†Perissodactyls	24	110	0.357	0.509
Gaubert et al. (2005)	$^{\ast}$ Feliformia	44	261	**0.003**	0.293
Gentry (1992)	$^{\ast}$ Bovidae	27	110	0.222	0.555
Gheerbrant et al. (2005)	†Proboscidae	12	113	0.424	0.990
Giannini and Simmons (2005)	$^{\ast}$ Pteropodidae	49	165	0.548	0.782
He et al. (2012)	$^{\ast}$ Erinaceidae	12	73	**0.001**	**0.002**
Kielan-Jaworowska and Hurum (2001)	†Allotheria	11	51	**0.002**	**0.018**
Ladevèze et al. (2010)	†Condylarthra	12	57	**0.013**	0.236
Lecompte et al. (2002)	‡Muridae	18	37	0.204	**0.014**
Ni et al. (2013)	$^{\ast}$ Primatomorpha	51	893	**0.001**	**0.036**
O’Leary et al. (2003)	$^{\ast}$ Theria	46	2200	**0.001**	**0.004**
Olivares and Verzi (2014)	$^{\ast}$ Echimyidae	21	59	0.624	0.255
Oliveira et al. (2011)	$^{\ast}$ Didephinae	51	80	0.261	0.225
Prideaux and Warburton (2010)	$^{\ast}$ Macropodidae	25	81	0.183	0.645
Sánchez-Villagra et al. (2006)	$^{\ast}$ Talpidae	17	152	**0.028**	0.253
Silcox et al. (2009)	†Apatemyidae	21	220	**0.001**	0.359
Spaulding et al. (2009)	$^{\ast}$ Artiodactyla	50	502	**0.001**	0.371
Springer et al. (1997)	$^{\ast}$ Marsupials	16	83	**0.001**	0.172
Steppan (1993)	$^{\ast}$ Phyllotini	49	82	0.058	**0.012**
Strait and Grines (2004)	‡Hominoidia	14	103	0.163	0.756
Thorington et al. (2002)	$^{\ast}$ Pteromyinae	20	73	**0.001**	0.132
Tomiya (2011)	$^{\ast}$ Carnivora	47	86	**0.001**	0.287
Weksler (2006)	$^{\ast}$ Oryzomyini	52	64	0.656	0.333
Wroe and Musser (2001)	$^{\ast}$ Thylacinidae	25	70	**0.019**	0.609
Zrzavý and ŘIčánková (2004)	$^{\ast}$ Canidae	32	77	**0.007**	0.082
	Totals	1234	7403	$21/40 <0.05$	$11/38<0.05$

*Note*: Bold values, $P < 0.05$.

$^{\ast}$ Data sets for which molecular trees were available.

†, ‡Data sets for which molecular data were unavailable: either because the ingroup were largely fossil taxa (b) or failed to meet minimum criteria following editing (c), for example, number of taxa, ratio of osteological to dental characters.

### Homogeneity of Signal

Application of the ILD test (Farris et al. 1995a, 1995b) found significant ($P <0.05$) heterogeneity between the osteological and dental character partitions in 21 out of 40 data sets, and a highly significant difference overall (Fisher’s combined probability $P = 4 \times 10^{-29}$, Table 1). Applying more stringent thresholds for significance still found widespread heterogeneity between osteological and dental partitions (17 out of 40 data sets had $P < 0.01$, and 12 out of 40 had $P < 0.001$). Using the tree-to-tree distance based test (IRD; Mounce et al. 2016) also identified widespread heterogeneity between dental and osteological partitions (11 out of 38 data sets have $P <0.05$, Fisher’s combined probability $P = 1\times 10^{-8})$.

### Relative Molecular Consistency

**Figure 2.**
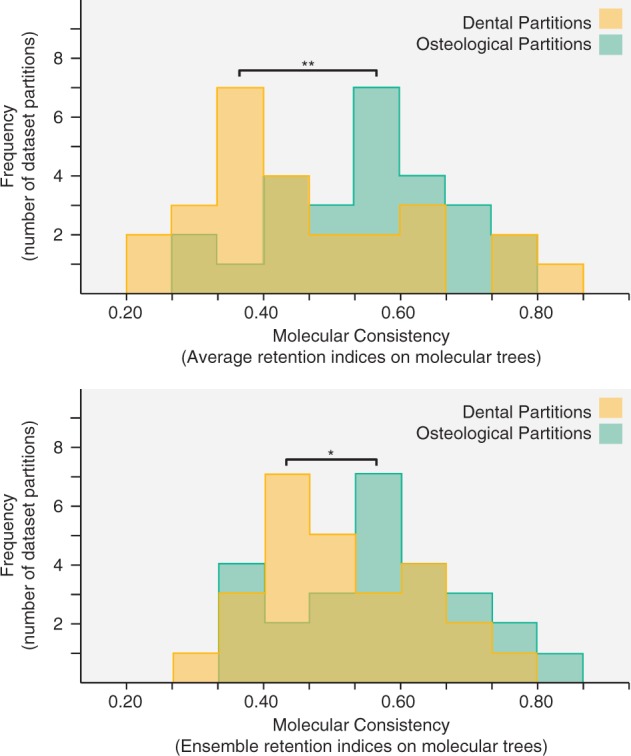
Tests for molecular consistency of morphological characters found significant differences between osteological and dental partitions (paired $t$-test, $P = 0.003$ for average retention indices, ri; paired $t$-test, $P = 0.036$ for ensemble retention indices, RI).

Molecular data are unavailable for almost all extinct taxa [an exception here being *Thylacinus* from Wroe and Musser (2001)]. Following the removal of fossil taxa, and the characters subsequently rendered uninformative, 14 morphological data sets were omitted from molecular comparisons (of which, 10 were composed largely of fossil taxa and 4 failed to meet the minimum dimensions for inclusion following taxon and character removal). This resulted in 26 data sets (Table 1) for which molecular data were available (seven of which molecular data were drawn directly from the same study as the morphological data). The subset of morphological matrices with molecular data available comprised a combined total of 698 taxa and 5589 morphological characters, and like the total data set, it also showed significant partition heterogeneity (Fisher’s combined probabilities of $P< 2 \times 10^{-14}$ for ILD tests and $P <5 \times 10^{-6}$ for IRD tests). More importantly, osteological characters were found to have significantly higher retention indices than dental characters when optimized onto molecular trees (Fig. 2). This difference was significant both for average retention indices of individual characters (paired $t$-test, $P = 0.003$) and for ensemble retention indices for whole partitions (paired $t$-test, $P = 0.036$). Furthermore, the difference was significant irrespective of the tree building methods that were used to infer molecular trees (i.e., maximum likelihood vs. maximum parsimony; see Supplementary Material available on Dryad). We note that it was not necessary to control for differences in numbers of characters in the dental and osteological partitions, using, for example, a jackknifing approach. While the Retention Index of most parsimonious trees is sensitive to differences in data set dimensions, our approach here was to optimize characters onto an existing tree (the molecular) in each case. The retention index (ri) for a given character is then the same regardless of whether this is calculated as part of the original partition, as part of a jackknifed subsample of this partition, or as a singly optimized character.

### Recovery of Nodes by Dental and Osteological Character Partitions

**Figure 3.**
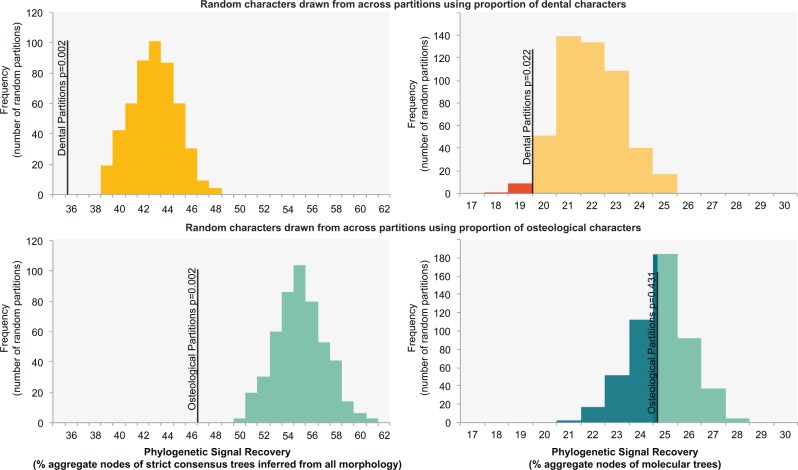
Results of node recovery tests showing distribution of phylogenetic signal recovery using either total morphological signal as a benchmark (left, percentage aggregate nodes of strict consensus trees inferred using both osteological and dental characters recovered) or molecular data as a benchmark (right, percentage aggregate nodes of molecular trees recovered). Characters were drawn from across both partitions using either the proportion of dental characters (above) or the proportion of osteological characters (below). The node recovery of the original partitions (i.e., dental or osteological, black lines) is mapped onto the distributions of equivalent randomly sampled partitions with estimated $P$ values (using proportion of random replicates recovering less signal recovery).

The node recovery test (Sansom andWills 2013) found that strict consensus trees inferred from exclusively dental characters recovered only 36% of the nodes of strict consensus trees inferred using all morphological characters as aggregated across data sets. This is a significantly lower percentage than strict consensus trees inferred using the same number of characters as dental characters, but drawn at random from across both partitions (median of 43% of aggregated nodes across all studies) ($P = 0002$, Fig. 3, see Supplementary Material available on Dryad). Strict consensus trees recovered using only osteological characters also recovered significantly fewer of the original strict consensus nodes compared to the searches using the same number of characters drawn at random from across partitions (47% of aggregated nodes for osteological only searches vs. median of 56% from random partitions of the same size; $P = 0.002$, see Supplementary Material available on Dryad). The modified node recovery test using molecular trees as a baseline for signal recovery yielded slightly different results. Strict consensus trees from searches using both osteological and dental characters of taxa for which molecular data were available recovered 29% of the nodes of molecular trees (as aggregated across data sets). Strict consensus trees from searches using only dental characters of these same taxa recovered just 19.8% of the molecular trees nodes; this is a significantly lower percentage of molecular tree nodes than strict consensus trees inferred using the same number of characters drawn at random from across both partitions (median of 22.2% of aggregated molecular nodes; $P = 0.022$; Fig. 3; Supplementary Material available on Dryad). However, strict consensus trees recovered using only osteological characters fell within the distribution of molecular node recovery seen in equivalent searches using the same number of characters drawn at random from across partitions (25.0% of aggregated molecular nodes for osteological searches vs. median of 25.2% from random partitions of the same size; $P = 0.431$).

## DISCUSSION

Within morphological data sets, osteological and dental character partitions exhibit significant heterogeneity as evidenced from both length-based tests (ILD) and tree-to-tree distance based tests (IRD). This indicates that a different phylogenetic signal is contained within each morphological partition; however, these tests alone do not indicate whether partitions differ in their ability to capture evolutionary history. Since it is impossible to know relationships with certainty, we used congruence with a qualitatively different and operationally distinct data source—namely molecules—as a benchmark of phylogenetic fidelity. Of the two morphological partitions, dental characters are a significantly poorer fit with independent molecular trees (Fig. 2). We interpret this to indicate that teeth are relatively less phylogenetically informative, which bears out predictions from developmental studies (Kangas et al. 2004; Harjunmaa et al. 2014) but contradicts findings from smaller scale empirical studies (Sánchez-Villagra andWilliams 1998; O’Leary et al. 2003;Williams 2007). That dental characters are less congruent with molecular data is unfortunate from a palaeontological perspective given that teeth constitute so much of the mammalian fossil record. Here we demonstrate that the application of simplified paleontological filters (the removal of osteology in node recovery tests) yields a loss of signal far in excess of the amount predicted from random incompleteness (Fig. 3). A similar pattern was found in an analysis of a single primate data set by Pattinson et al. (2015) whereby “artificial fossil templates” using largely dental characters were found to result in appreciably less signal recovery than “random templates”. Indeed, they state that “for most fossils in our data set known for fewer than 180 sampled characters (i.e., >50% missing data), taphonomically induced bias decreases phylogenetic accuracy” (Pattinson et al. 2015, p. 179). Their “random templates” are akin to the random missing data analysis used in our study (unlike their “random states” that we interpret as providing a baseline of node recovery for phylogenetically random data with the same general properties of state frequencies). Pattinson et al. (2015) found that sampling across morphological partitions performed better than sampling predominately from a single partition and interpreted this as supporting the principle that characters should be sampled from as many sources as possible (Kluge 1989; O’Leary et al. 2003; Mounce et al. 2016). However, the empirical distribution of characters was such that artificial templates dominated by one partition were always dominated by dental characters. As such, insufficiency of a single partition equates to insufficiency of dental characters in this case. In our meta-analyses of 40 data sets (26 with molecular trees), we have explicitly tested both dental only partitions and osteology only partitions. Both dental only and osteology only partitions recover significantly less signal (percentage of original strict consensus nodes when using all morphology as benchmark) than partitions of equivalent size that sample characters randomly from across partitions. This in itself would imply that characters should be sampled widely from across both partitions. When molecular trees are used as a benchmark of signal recovery, however, dental only searches are found to perform significantly worse than random partitions of equivalent size, whereas osteological only searches do not (Fig. 3). Application of molecular data, therefore, indicates that dental data are generally less-reliable indicators of phylogenetic history than osteological data. Not only do dental characters exhibit elevated levels of relative homoplasy, but they are also unlikely to be sufficient for accurate phylogeny reconstruction on their own.

When results from different studies are compared, a coherent picture emerges. Dental morphology is found to convey a phylogenetic signal that is different to that derived from osteology and is comparatively less consistent with molecular data. That dental characters are more homoplastic accords with the observation that they are oversaturated (Dávalos et al. 2014), as well as with developmental evidence for the relative ease and correlation of shape changes in teeth (Kangas et al. 2004; Harjunmaa et al. 2014). Furthermore, loss of osteology (the common palaeontological case) causes significant and disproportionate degradation of phylogenetic signal, such that analyses of dental morphology alone are less likely to reconstruct relationships accurately. Notwithstanding all of the above considerations, we do not advocate the wholesale exclusion of dental morphology from phylogenetic and evolutionary studies. Not only would that exclude many taxa known only from teeth, but also neglect the reliable phylogenetic signal that must exist in at least some dental characters, as well as the hidden support that may exist between dental and other characters (Gatesy et al. 1999); the inclusion of dental characters alongside osteological recovers 29% of molecular tree nodes compared to 25% when using osteology only. Instead, it is necessary to identify subsets of morphology that are less subject to convergence and to directly address the issue of oversaturation and nonindependence (Dávalos et al. 2014) [e.g., by identifying suites and cliques of convergent or correlated characters (O’Keefe and Wagner 2001; Holland et al. 2010)]. The phenomenon appears to be widespread for all mammals; it is possible that it could extend to other vertebrates, although the condition of heterodonty in mammals may have compounded the issue, potentially through failure to properly account for serial homology. In all instances, we strongly advocate meta-analyses of cladistics data sets; it is only when data from a wide diversity of clades are comprehensively sampled and compared that large-scale patterns such as these become apparent (Sansom andWills 2013; Mounce et al. 2016).

## SUPPLEMENTARY MATERIAL

Data available from the Dryad Digital Repository: http://dx.doi.org/10.5061/dryad.k23mq.
